# Hierarchically Nanostructured CuO–Cu Current Collector Fabricated by Hybrid Methods for Developed Li-Ion Batteries

**DOI:** 10.3390/ma11061018

**Published:** 2018-06-15

**Authors:** Jin-Young So, Chan-Ho Lee, Ji-Eun Kim, Hyun-Jee Kim, Joonha Jun, Won-Gyu Bae

**Affiliations:** Department of Electrical Engineering, Soongsil University, Seoul 156-743, Korea; igiyoun@ssu.ac.kr (J.-Y.S.); chlee9306@gmail.com (C.-H.L.); wldms5103@gmail.com (J.-E.K.); qmffptmb@gmail.com (H.-J.K.); joonha9974@gmail.com (J.J.)

**Keywords:** hierarchical current collector, laser ablation, oxidation, CuO, imprinting

## Abstract

We present a simple method of fabricating a hierarchically nanostructured CuO–Cu current collector by using laser ablation and metal mold imprinting to maximize the surface area. The laser ablation of the Cu current collector created the CuO nanostructure on the Cu-collector surface. The microstructure was transferred by subsequent imprinting of the microstructure metal mold on the Cu collector. Then, the laser-ablation nanostructure was formed. Consequently, a hierarchical structure is generated. The laser-ablated hierarchical CuO–Cu current collector exhibited an improved capacity while maintaining a cyclability that is similar to those of conventional graphite batteries.

## 1. Introduction

With the rapid development of technology, the demand for various mobile electronic products such as cell phones, tablets, and smart healthcare devices has increased explosively [1,2,3], and studies with the aim of developing lithium-ion batteries (LIB) of a high efficiency, stability, and low cost have been actively conducted. Graphite (372 mAh g^−1^) [4] has been used as the anode material in the fabrication of Li-ion batteries with a low theoretical capacity, because the anode material directly affects the capacity of Li-ion batteries. Although silicon-based material is emerging as an ideal replacement for the commercial carbonaceous material owing to its highest theoretical capacity (4200 mAh g^−1^), it would be difficult to replace that because of the volume expansion of the silicon-based anode during the charge/discharge process. Innovative silicon nanostructures, therefore, have been used in numerous studies in order to prevent volume expansion of Si-based anodes (i.e., nanoparticles [5,6], nanowires [7], and nanotubes [8]). Despite the important achievements mentioned, poisonous and dangerous reagents are not appropriate in a synthesis process. When sophisticated equipment is necessary or expensive and multi-stage synthesis is adopted, it is not easy to increase the scale. Accordingly, there still remain significant challenges to overcome the commercialization of the Si anode.

In the case of transition metal oxides, the capacity is much higher than the capacity of the graphitic carbon, and the volume change is not as great as the silicon material. Therefore, the use of a transition metal oxide as the anode material appears to be an appropriate way to overcome the drawbacks of the alloy anode material [9]. Copper (Cu)-based oxides such as copper (Cu_2_O) and copper (CuO) oxides, as well as transition metal oxides, are under the spotlight for promising candidates for use in LIB, solar cells, supercapacitors, gas sensors, biosensors and catalysts due to their inherent properties as p-type semiconductors with a low band gap energy, high optical absorption and high catalytic activity.

Cupric oxide (CuO) [10] and cuprous oxide (Cu_2_O) [11], with the respective capacities of 674 mAh g^−1^ and 375 mAh g^−1^, have received enormous attention owing to high theoretical capacities, low costs, non-toxicity, and abundant natural reserves. Alternatively, regarding the LIB for which CuO and Cu_2_O serve as the anode materials, the generation of very large volume expansions occurs during the battery lithiation and delithiation processes, resulting in not only a separation from the copper (Cu) current collector, but a poor cyclability, low capacity, and low electronic conductivity [12,13]. To solve these problems, numerous studies have been conducted to mitigate the volume-expansion stress, where the anode material nanostructure has been fabricated into nanoparticles, nanowires, nanotubes, nanorods and nanoflake structures [14,15,16,17]. Notwithstanding the significant improvements in the electrochemical performance of the Li-ion batteries that are enabled by these studies, the corresponding methods require exorbitant devices, complicated preparations, and time-consuming tasks, and difficulties of low-cost large-area processing are encountered as well.

Meanwhile, fabrication of nanostructured metal oxides generated on the metal substrates by fast pulsed laser ablation has been reported. A series of nanostructured metal oxides have been synthesized by pulsed laser ablation of metal substrates.

Here, graphite, the anode material of the conventional battery, was replaced with CuO through the formation of CuO on the Cu surface that was achieved by a simple, fast, innocuous, and inexpensive laser irradiation of the Cu current collector; nanostructures, which were made through laser ablation, prevented the degradation of the battery capacity and cyclability that are results of the volume expansion. The laser ablation spontaneously allowed the formation of the nanostructure on the Cu current collector. Then, the microstructure was transferred onto the Cu current collector through the imprinting of the microstructural metal mold on the Cu current collector, wherein the nanostructure was formed. This process was carried out considering the use of a hierarchical structure [18] consisting of both the nano- and microstructures, which accommodate volume expansion of Si more than solely the nanostructure. The lithium-ion battery that is based on the hierarchically nanostructured CuO–Cu current collector with the micro- and nanostructures not only maintained a cyclability that is similar to those of the conventional types, but the capacity was improved twofold. The proposed method of this paper can be directly applied to the existing battery-fabrication process, as laser ablation enables an increase in the battery capacity via a chemical-free process in a fast and simple manner.

## 2. Materials and Methods

### 2.1. Laser Ablation

A single-mode HY-FM20 nanosecond (ns) pulsed laser with output powers of 10, 20, and 30 W at room temperature and in air was used in this study to create the multiscale structure on the surface of the Cu current collector. The laser beam of the YLP-V2-model HY-FM20 laser source (IPG Lasers, Burbach, Germany) generates a central-emission wavelength of 1064 nm, and the maximum pulse-repetition rate of 1000 kHz was delivered by a total of three reflective mirrors, a beam expander, an *x*- and *y*-axes galvanometer scanner, and a telocentric focusing lens. Moreover, the beam-spot size at the focal point was approximately 10 μm. The sample movement was generated by an *x*-/*y*-/*z*-axes movable stage with the ball-screw mechanism. The nanosecond laser was used to ablate over the Cu current collector (30 mm × 30 mm) at the working speed of 2000 mm/s, the width of 0.01 mm, the moving speed of 500 mm/min, and the power of 100 W. 

### 2.2. Metal Mold

The metal mold (30-mm length × 30-mm width × 2-mm thickness) with the line-pattern (500-μm wavelength) was fabricated using wire electrical discharge machining (WEDM) with a precision process. The WEDM process was used to process the master metal mold, the rigidity of which was high. For the WEDM process that was used in the metal precision processing, a method to apply a square-wave alternating current (ac) voltage between the wire electrode and the workpiece was employed [19]. In the present study, the master–mold surface was cut using electrical-discharge energy via the positive voltage of +126 V, the negative voltage of −80 V, the positive duration of 28 µs, and the negative duration of 50 µs, for which the commercial EZ20S WEDM machine (Seoul Precision Machine, Seoul, Korea) was employed.

### 2.3. Imprinting Process

The pattern of the fabricated metal mold was transferred to Cu foils (Hohsen Corporation, Osaka, Japan) that subsequently served as current collectors using the imprinting process [20]. The imprinting process was performed by pressing the patterned metal mold onto the Cu foils under a pressure of 10 MPa for 5 min. 

### 2.4. Electrochemical Measurements

A scanning electron microscope (SEM)–electrochemical dispersive spectroscopy (EDS) investigation was carried out using the JSM 5900 SEM (JEOL, Tokyo, Japan) equipped with the X-ray-microanalysis Oxford-ISIS EDS (Oxford Instruments, Oxford, UK) that comprises the silicon (lithium) detector. The electron-beam voltage for the observation was between 5 kV and 20 kV and its current is between 0.01 nA and 10 nA. The samples were analyzed using the EDS instrument under a low vacuum, and with the working distance of 10 mm, the beam voltage of 20 kV, and the current of 0.25 nA.

Electron probe microanalysis (EPMA)–wavelength dispersive spectroscopy (WDS) maps were derived using the JXA 8800 R SUPER PROBE electron microprobe (JEOL, Tokyo, Japan) equipped with four spectrometers. The electron-beam voltage was 15 kV and the current was 40 nA. Cu, carbon, and oxygen maps were performed using the sample, and the maps were obtained with sets of 300 × 300 spots on areas of approximately 600 × 600 µm^2^. In every map, each color represented the corresponding element, and every color was independent of the other colors. 

### 2.5. Electrochemical Properties of Graphite and CuO Electrodes

The electrochemical properties of the anode electrodes were evaluated using size-2032 coin-type cells (Hohsen Corp., Osaka, Japan). The cell assembly was carried out in an argon (Ar)-filled glove box (<5 ppm of H_2_O and O_2_). The as-prepared anode electrodes, cut with a diameter of 13 Ф, and lithium foil, were used as the working and counter electrodes, respectively. Celgard 2400 porous polyethylene (Celgard, Cheongju-Si Chungbuk, Korea) and 1.1-M lithiumhexafluorophosphate (LiPF_6_) dissolved in ethylene carbonate (EC):dimethyl carbonate (DEC) of a 1:1 vol % were used as the separator and the electrolyte, respectively. The charge–discharge characteristic curves of the assembled cells were obtained using the WBCS3000L multichannel battery tester (WonATech, Seoul, Korea) at 25 °C. Electrochemical impedance spectroscopy (EIS) was performed using the Eco Chemie electrochemical analyzer (Metrohm, Utrecht, The Netherlands) at the applied voltage of 5 mV in the frequency range of 10–100 kHz.

## 3. Results and Discussion

**Figure 1:**
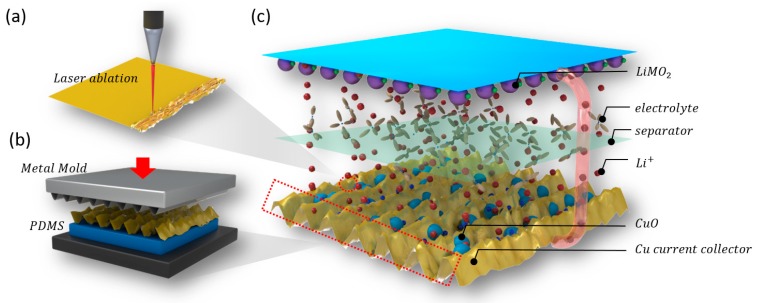
Fabrication of the hierarchically nanostructured CuO–Cu current collector: (**a**) CuO–Cu current collector with the nanostructure formed from the laser ablation on the Cu current collector; (**b**) imprinting method over the CuO–Cu current collector for the microstructure addition; (**c**) lithium-ion battery for which the hierarchically nanostructured CuO-Cu current collector was applied.

Figure 1 illustrates the overall fabrication process of the hierarchically nanostructured CuO–Cu current collector and its application in the design of the lithium-ion battery. The first step, as shown in Figure 1a, represents the nanosecond laser ablation on the Cu current collector; The surface of the Cu collector in the laser-cutting process was immediately vaporized by high energy and thermal laser pulses. The avalanche ionization induced by the intense laser field excited the copper vapor to the plasma. Plasma and/or superfine Cu particles were then oxidized in air and deposited on the surface of the Cu current collector substrate and cooled to room temperature within a very short time. Finally, due to the very high pressure of the atmospheric atmosphere in which the superfine particles were generated, a uniformly distributed faulty CuO hierarchical nanostructure which was firmly adhered to the surface of the Cu current collector after the CuO particles retracted onto the substrate. The Cu was evaporated by the heat generated and converted into CuO through the two-step oxidation of 4Cu + O_2_ → 2Cu_2_O, 2Cu_2_O + O_2_ → 4CuO in air [21]. 

The second step of Figure 1b shows the imprinting of the line-patterned metal mold on the Cu current collector with the nanostructure constructed by the laser ablation. The sinusoidal pathway of the proposed line-patterned metal mold obtained the 500-μm wavelength. Then, the Cu current collector in which the nanostructure was formed was placed on polydimethylsiloxane (PDMS), and the line pattern was transferred onto the Cu current collector using imprinting by the generated line-patterned metal mold (under the pressure of 10 MPa for 5 min). The PDMS served as the buffer layer and allowed the microsize line pattern to be properly transferred to the Cu current collector while the ablation-produced nanostructure was entirely maintained. The CuO–Cu current collector was consequently fabricated with a hierarchical structure that is frequently used to broaden the surface area in nature.

The last step, shown in Figure 1c, is the demonstration of the application of the hierarchically nanostructured CuO–Cu current collector, which replaced the conventional Cu current collector and the graphite anode material, in the design of the lithium-ion battery. The cycling-performance test was subsequently carried out.

**Figure 2:**
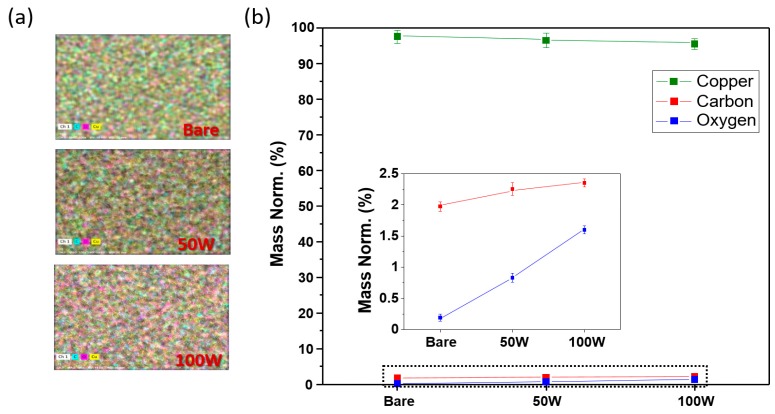
Electron probe microanalyzer (EPMA) and electron dispersive spectroscopy (EDS) data of copper, carbon, and oxygen on the Cu current collector after the laser ablations of 50 W and 100 W: (**a**) EPMA map; (**b**) EDS analysis.

Figure 2 shows the EPMA and EDS chemical analyses regarding the CuO–Cu current collector, which was formed as a result of the laser ablation on the bare Cu current collector. In Figure 2a, the top sections of each of the EPMA images indicate the regions on the bare Cu current collector under the 50-W and 100-W ablation powers. As the analysis data of the bare Cu current collector show, at the top of the image the oxygen component of the pink region appears to be low, and then it gradually increases further down the image; that is, as the Cu current collector was oxidized by the laser’s high energy and heat, the CuO became evenly generated on the surface. The EDS numerical-analysis data are shown in Figure 2b, where the graph indicates the relative contents of Cu, C, and O_2_ as quantitative materials; moreover, the inset graph, an enlargement of the C and O_2_ results, demonstrates that the bare Cu current collector consisting of 97.80% of Cu and 0.22% of O_2_ represents significantly lower oxygen content compared with the Cu content. Alternatively, the Cu current collector at 100 W comprising 96.94% of Cu and 0.81% of O_2_ is the result of the state at 50 W consisting of 96.09% of Cu and 1.55% of O_2_, respectively, and this presents the rapid O increase. This result shows the combining of the Cu and O_2_ in the air and the subsequent deposition of this combination on the surface that increased the oxygen floating on the surface. The Cu current collector that was ablated with the 100-W laser was used in the subsequent experiments, where the efficiency was increased as the CuO content grew. Furthermore, Figure 2 shows that laser ablation can quickly and simply produce CuO with a high theoretical capacity without any chemical treatment, and a higher capacity that outperforms the conventional lithium-ion battery is anticipated.

**Figure 3:**
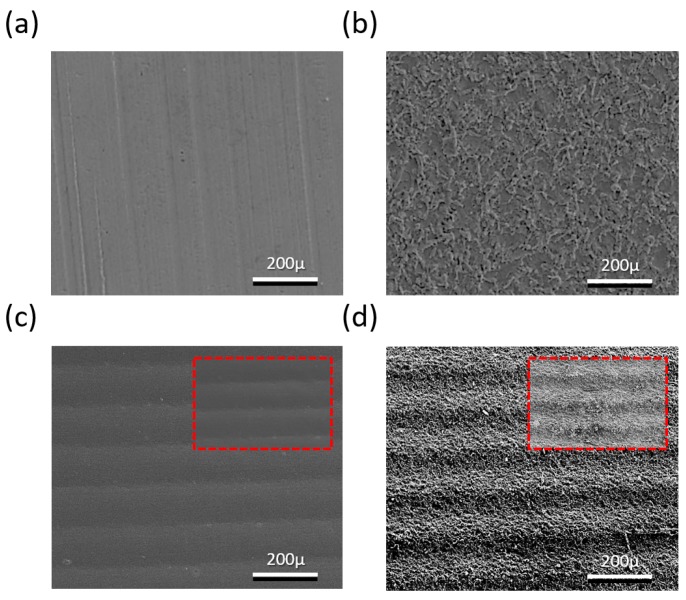
Scanning electron microscope (SEM) images of four samples of the Cu current collector: (**a**) bare Cu current collector; (**b**) laser-ablated Cu current collector; (**c**) imprinted Cu current collector; (**d**) laser + imprinted Cu current collector.

SEM (500×) images of the bare Cu current collector, laser-ablated Cu current collector, imprinted Cu current collector, and laser + imprinted Cu current collector are represented in Figure 3a–d, respectively. A flat surface is seen in Figure 3a, while in Figure 3b, the surface becomes very rough due to the explosion of the laser energy. Figure 3c shows the illustration of the sinusoidal pathway of the Cu current collector of the 500-μm wavelength that was constructed using the imprinting process. Figure 3d shows the laser-ablated nanostructure and the combined-hierarchy Cu current collector with the microstructure that was formed using metal-mold imprinting.

**Figure 4:**
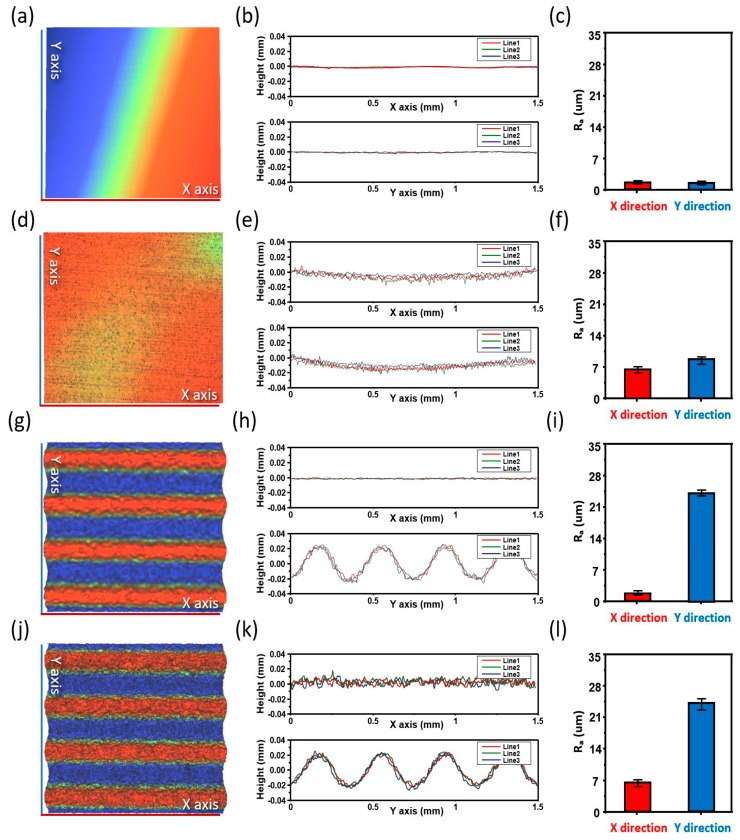
Three-dimensional (3D) image: (**a**,**d**,**g**,**j**); line-scan profiles in the *x* and *y* directions: (**b**,**e**,**h**,**k**); surface-roughness averages (R_a_): (**c**,**f**,**i**,**l**). form above the first row, bare Cu current collector, Cu current collector after the laser ablation (laser-ablated Cu current collector), Cu current collector after the imprinting metal mold, and Cu current collector after the laser ablation + imprinting sequentially.

Figure 4a,d,g,j respectively show the three-dimensional (3D) surface-analysis images of the four types of the fabricated Cu current collector, as follows: bare Cu current collector, laser-ablated Cu current collector, imprinted Cu current collector, and laser + imprinted Cu current collector. The figure shows that 3D images provide results that are the same as those of Figure 3. The line-scan profiles of the bare Cu current collector, laser-ablated Cu current collector, imprinted Cu current collector, and laser + imprinted Cu current collector, measured using the non-contact 3D microtiter, are shown in Figure 4b,e,h,k, respectively. The graphs above and below show the line-scan profiles of the Cu current collectors that were extracted at three different spots along the *x*- and *y*-axes, respectively. The line-scan profile indicates the elevation of the surface relative to zero. Also, the greater the amount of ripples, the rougher the surface and the wider the surface area; these results also indicate that the surface is flat, which is shown by the *x*- and *y*-axes on the line-scan profiles where only straight lines are apparent.

Moreover, as illustrated in Figure 4e, the line-scan profile comprises many ripples on the *x*- and *y*-axes relative to zero. Accordingly, the surface area per unit area of the proposed Cu current collector became larger than that of the bare Cu current collector due to the spontaneously generated laser-ablation ripple. From the line-scan profile of Figure 4h, the 500-μm wavelength occurred periodically, thereby resulting in an effectively performed transfer of the metal-mold pattern and the incremental transfer of the surface area per unit area compared with the bare Cu current collector. Figure 4k, in contrast, shows the line-scan profile with numerous ripples and periodic appearances of the μm wavelengths that are owing to the simultaneously presented laser-ablation nanostructure and the metal-mold microstructure. This result represents a nanostructure that is not affected by the imprinting, a well-transferred microstructure, and a hierarchical structure that became the widest surface area per unit area.

Quantitative evaluation was performed to compare the increase of the surface area accurately. Figure 4c,f,i,l show the surface-roughness averages (R_a_) of the bare Cu current collector, laser-ablated Cu current collector, imprinted Cu current collector, and laser + imprinted Cu current collector, respectively. The R_a_ values indicate the degree of the surface roughness, where the value increases with the increasing roughness of the surface; that is, the incremental increase of the R_a_ value compared with the previous value means that the surface area per unit area has increased [22]. Furthermore, the bar values along the *x*- and *y*-axes and the standard error demonstrate the mean and the standard deviation of the red, green, and blue along the *x*- and *y*-axes of the line-scan profiles shown in Figure 4b,e,h,k. The R_a_ values of the *x*- and *y*-axes in Figure 4c are 1.73 and 1.72, respectively. Compared with Figure 4c,f shows increases of the R_a_ values of the *x*- and *y*-axes (6.63 and 8.72) by 383% and 506%, respectively, thereby quantitatively confirming an enormous increase of the surface area. In Figure 4i, the R_a_ values of the *x*- and *y*-axes are 1.76 and 21.7, respectively, while the R_a_ value of the *x*-axis is similar to that of Figure 4c and the *y*-axis was increased by 1261%. As shown in Figure 4i, the R_a_ values of the *x*- and *y*-axes are 6.52 and 24.4, respectively. Here, the figures suggest that the surface area per unit area among the samples increased the most due to the increase of the R_a_ values of the *x*- and *y*-axes by 376% and 1418%, respectively, compared with Figure 4c. The results of Figure 3 and Figure 4 provide the optimal method for maximizing the surface area for which the hierarchically nanostructured structures were used.

**Figure 5:**
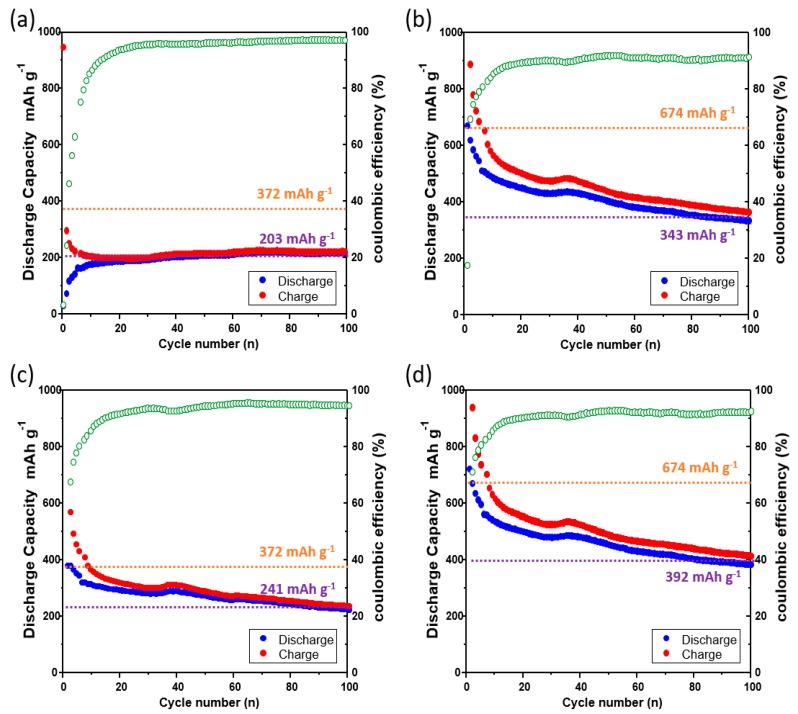
Charge–discharge graphs of the use of the four samples of the Cu current collector for the lithium-ion battery (orange dashed line: theoretical capacity, yellow dashed line: capacity after 100 cycles): (**a**) the charge–discharge curves of the bare Cu current collector when graphite is deposited on the current collector; (**b**) the charge–discharge curves of the CuO (the product of the oxidation of the Cu current collector by the laser)-deposited Cu current collector irradiated with the laser; (**c**) the charge–discharge curves of the graphite-deposited Cu current collector imprinted with the microstructure metal mold; (**d**) the charge–discharge curves of the CuO (the product of the oxidation of the Cu current collector by the laser)-deposited Cu current collector irradiated with the laser and imprinted with the microstructure metal mold.

Figure 5 shows the charge–discharge curves of the lithium-ion batteries that were prepared using the bare Cu current collector, laser-ablated Cu current collector, imprinted Cu current collector, and laser + imprinted Cu current collector. Graphite was used as the anode material of the bare Cu current collector and the imprinted Cu current collector. Alternatively, CuO, deposited on the surfaces of the laser-ablated Cu current collector and the laser + imprinted Cu current collector as the product of the laser oxidation of the Cu current collector, was used as the anode material. The charging and discharging were carried out at the current density of 6 A g^−1^ in the range of 0.01–1.50 V.

Figure 5a shows the charge–discharge curves of the lithium-ion battery of the bare Cu current collector. The capacity after 100 cycles is 203 mAh g^−1^. The initial coulombic efficiency (CE) of Figure 5a was maintained at 98% after 25 cycles, where the CE signifies the battery efficiency [23]. Figure 5b illustrates the charge–discharge curves of the lithium-ion battery of the laser-ablated Cu current collector. The capacity after 100 cycles is 343 mAh g^−1^. The CE was maintained at 92% after 25 cycles. The capacity after 100 cycles increased by 168%, and the CE was analogously maintained in comparison with Figure 5a; this is because the capacity was increased as CuO, which was formed by the laser ablation, was used as the anode material. Moreover, the nanostructure, which was constructed by the laser ablation, created a void space. The void space not only provides space for CuO to accommodate the volumetric expansion during charging and discharging, but it also enlarges the surface area so that the increased interatomic attraction force enables the effective adherence between CuO and the Cu current collector; this leads to an improved conductivity and helps to improve the capacity as well. Figure 5c shows the charge–discharge curves of the lithium-ion battery of the imprinted Cu current collector. The capacity after 100 cycles is 241 mAh g^−1^. The CE was maintained at 95% after 25 cycles. The capacity after 100 cycles increased slightly by 119% compared with Figure 5a. This finding is because of the unaltered theoretical capacity that is due to the use of the same anode material, graphite, but also since the microstructural sinusoidal pathway increases the surface area, which may provide the positive impacts as it expands the surface area where graphite and lithium react. Therefore, the imprinted Cu current collector has better conductivity than the bare Cu current collector. Figure 5d demonstrates the charge–discharge curves of the lithium-ion battery of the laser + imprinted Cu current collector. The capacity after 100 cycles is 392 mAh g^−1^. The CE was maintained at 94% after 25 cycles. The capacity after 100 cycles was increased by 193% compared with Figure 5a, and the CE is analogous to Figure 4c. In comparison with the other samples, the capacity after 100 cycles is the highest. This finding is because of the micro and nano mixed structures that are used to increase the surface area in nature; that is, the surface area became the widest among the samples, thereby leading to the reduced separation between CuO and the Cu current collector that is due to the highest increase of the interatomic attraction.

## 4. Conclusions

This paper presents the proposal of a simple method for the fabrication of a hierarchically nanostructured CuO–Cu current collector for which laser ablation and a metal mold are used with respect to the Cu current collector. The disadvantage of the previous process regarding the application of graphite as the anode material of the Cu current collector for the conventional battery is the difficulty of large-area applications and a low theoretical capacity. In this study, however, laser ablation over the surface of the Cu current collector showed that it is possible to directly apply graphite as the anode material through the formation of CuO with a high theoretical capacity in a fast and simple manner. Furthermore, the laser energy spontaneously generated the nanostructure while oxidized Cu particles were deposited on the surface of the Cu current collector, thereby providing the void space to buffer the CuO volume expansion that occurred during the charging and discharging. In addition, the microstructure was transferred to the Cu current collector with the laser-ablation nanostructure using the metal mold together with the sinusoidal pathway for the production of the hierarchical structure. This hierarchical structure maximizes the surface area per unit area of the Cu current collector, thereby leading to an increase in the attraction force between the atoms that then increased adhesion between the Cu current collector and CuO, resulting in an increased battery capacity. The hierarchically nanostructured CuO–Cu current collector presented in this study can be applied directly in the conventional battery-manufacturing processes, and it can also increase the battery capacity and cyclability in an innocuous, chemical-free, quick, and simple manner.
